# Typhoid Fever and Smallpox

**Published:** 1847-11

**Authors:** 


*Typhoid Fever and Smallpox. By* M. SERRES.—On a former occasion we mentioned the treatment which M. Serres considers most efficient in typhoid fever, viz., the black sulphuret of mercury; and, also, how the profession was led to adopt this method, from the good effects of mercurial preparations on the eruption of smallpox. In another communication to the Academy of Sciences the learned professor continues the same subject.

The intercurrent affections, says M. Serres, two diseases progressing at the same time in the same individual, each preserves its physiognomy, its special characters, although the intimate nature of both may have undergone a considerable modification. The most severe of the two almost invariably communicates its gravity to the other, and diminishes if it does not destroy the chances of recovery. In the treatment, therefore, of intervening disorders, the most dangerous must exclusively occupy the attention of the therapeutist; in order to judge a curative method, it should be put to the test of these intercurrent affections, and to this test I have submitted the method I propose for the cure of typhoid fever; and have chosen the most severe complication—variola. The dangers of confluent smallpox are known to all; vaccination has considerably diminished them, but within certain limits. Thus, at the Hospital of La Pitié, during the epidemic of 1825, out of 162 vaccinated subjects attacked by the epidemic, twenty-five died. From the statistics published by Dr. Bousquet, it appears that in the thirty epidemics of smallpox observed in France between the years 1816 and 1841, 5963 vaccinated persons were attacked, and sixty-two died. Analogous results have been noticed in England, Germany, Italy, and Sweden. This revival of smallpox was attributed to the inefficiency of vaccination—a conclusion which we consider erroneous: "It is," continues M. Serres, "to the influence of intercurrent disorders that this impotency of vaccination to prevent variola should be attributed, and chiefly to typhoid fever. During the epidemic of 1825 we were struck principally by two facts: the first was the progress of the pustular eruption, which was the same in the vaccinated subjects as in those who had not been vaccinated; and the second was the presence in the intestines of the facial characters of typhoid fever. The epidemic, in one word, was double; it was at the same time variolic and typhoid. The mortal influence of typhoid fever in cases of smallpox has appeared to us still more evident in cases of non-vaccinated patients. We may ask ourselves if, before the discovery of vaccine, the interference of typhoid fever was not one of the great elements of the danger of variola; and we will find in the symptomatic history of ancient epidemics abundant proofs that such was the fact. Typhoid fever is not, therefore, a new disease, but smallpox and typhoid fever were, perhaps, coeval: the cutaneous eruption of the former struck more forcibly the observer, and concealed the complication. It would seem that, after Jenner's discovery, variola was in a great measure arrested, and typhoid fever, being unmasked, was at last recognised." After having established that in most cases the complication of typhoid fever was the principal source of danger in cases of smallpox, the learned professor proceeded to say that he had watched attentively the effects of the black sulphuret of mercury in such cases, and that, in proportion as the medicine relieved the symptoms of the typhoid fever, it also weakened the violence of the smallpox.—*Ibid.*

